# Supplementary material for: Structure of the prefusion-locking broadly neutralizing antibody RVC20 bound to the rabies virus glycoprotein
Source: Nat Commun. 2020 Jan 30;11:596. doi: 10.1038/s41467-020-14398-7 (PMC6992781; doi:10.1038/s41467-020-14398-7)
Supplement: Supplementary file 1 — Supplementary Information [file 41467_2020_14398_MOESM1_ESM.pdf]

Supplementary Information

# Structure of the prefusion-locking broadly neutralizing antibody RVC20 bound to the rabies virus glycoprotein

Hellert et al.

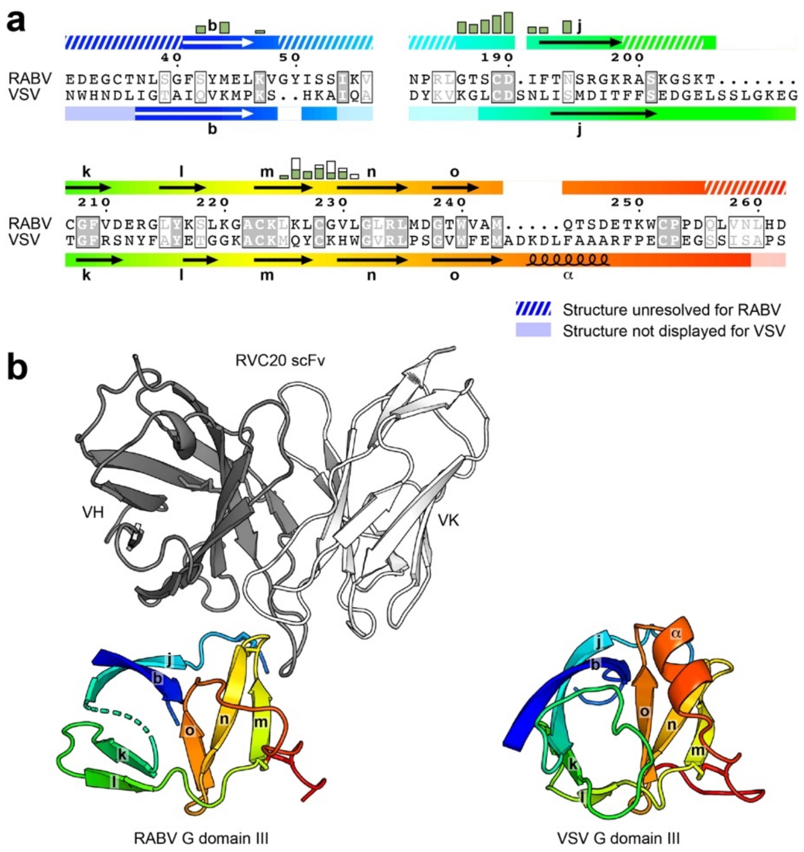


**Supplementary Fig. 1: Comparison of domains III between RABV G and VSV G.**
**a** Alignment of the RABV G residues in our domain III construct with the respective sequences from VSV G. β strands are shown as arrows labeled with lower-case letters in accordance with the complete ectodomain structure of VSV G[1]. Relative contributions to the RVC20 epitope by the heavy chain (green) and the light chain (white) are indicated as bars above the sequence as in Fig 1c. **b** Comparison of the domain III structures of RABV G (left) and VSV G (right, PDB: 5I2S). Their root-mean-square deviation (RMSD) is 3.8 Å over 64 residues.

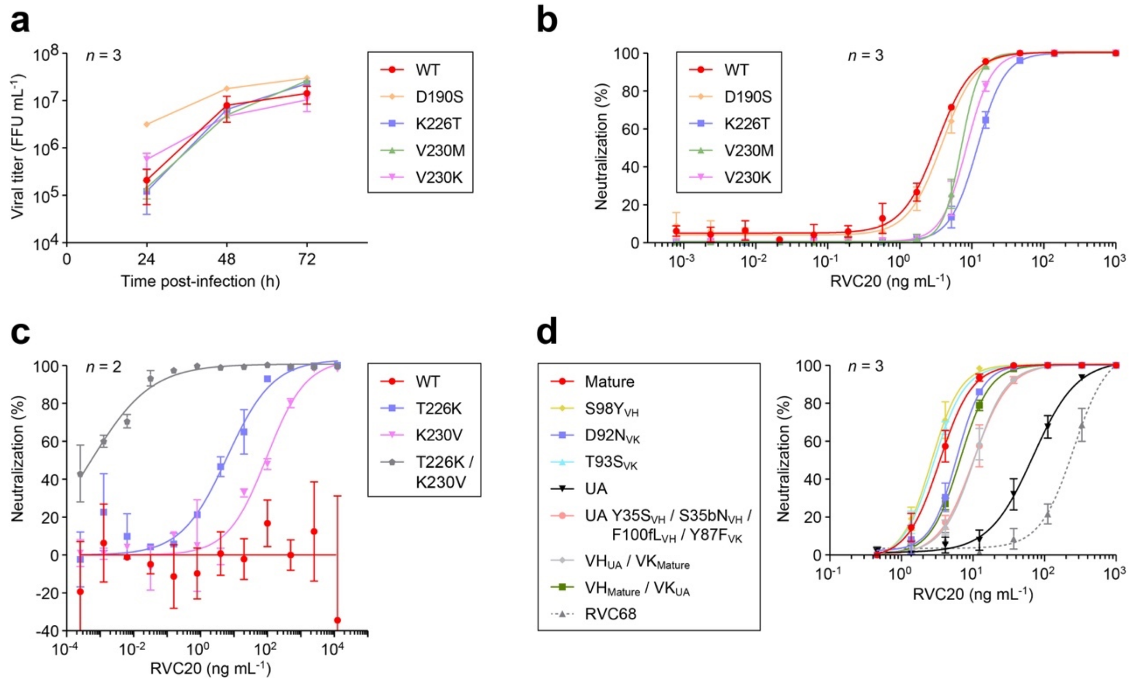


**Supplementary Fig. 2: Growth curves of recombinant viruses and neutralization curves.** **a** Recombinant RABV production assay in BSR cells infected with wild-type or mutant viruses (MOI = 0.1) for the indicated time points before virus titration in supernatants. **b** RVC20 neutralization of wild-type or mutant RABV on BSR cells 48 h after infection. **c** RVC20 neutralization of wild-type and mutant LBV G-pseudotyped lentiviruses with mAb RVC20 on BHK-21 cells 72 h after infection. **d** RVC20 variants neutralization of wild-type RABV on BSR cells 48 h after infection. The number *n* of independent experiments is indicated for each graph. Data are displayed as means ± s.d. Source data are identical to those for Figures 1e,f & 2c in the Source Data file.

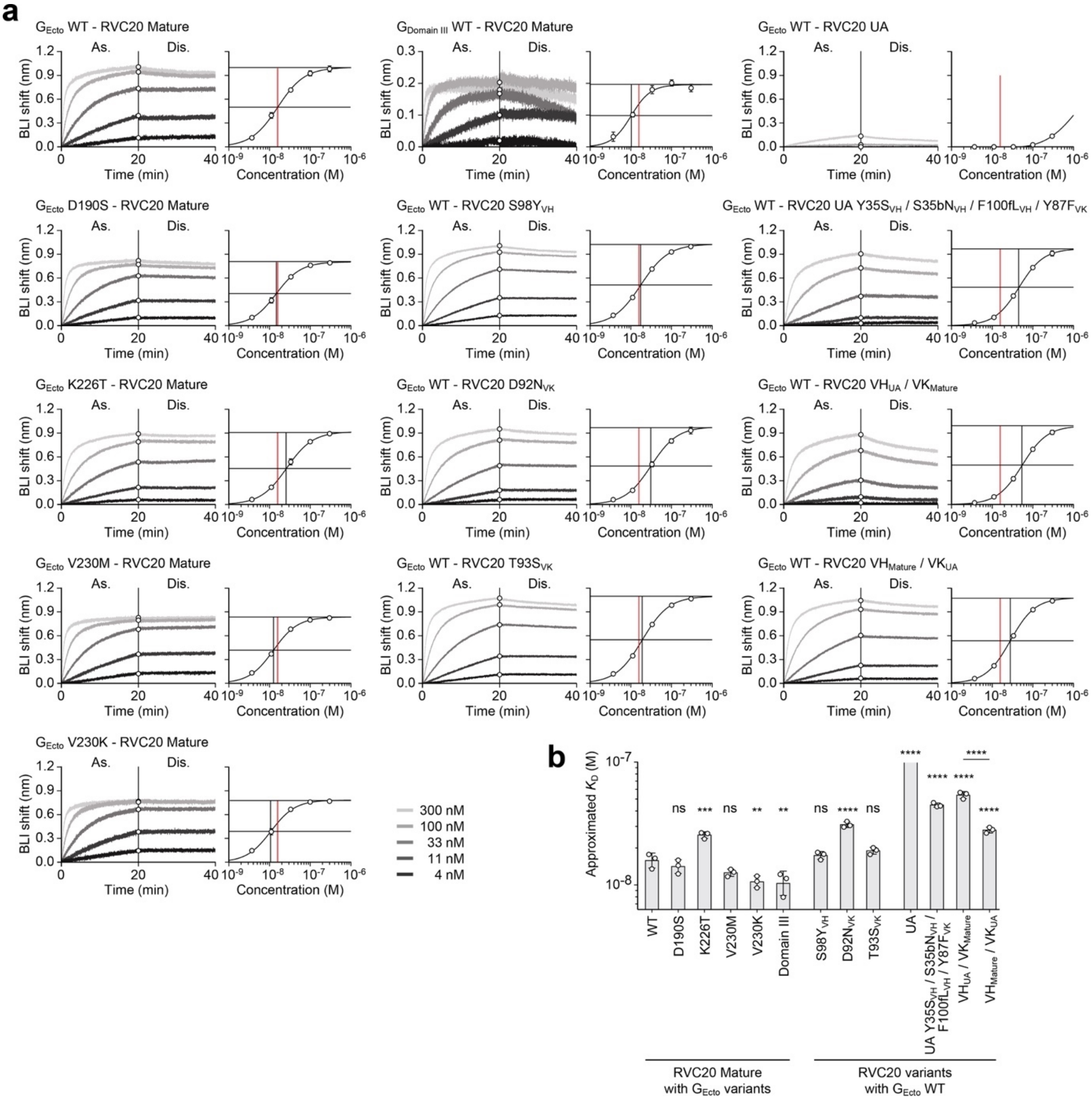


**Supplementary Fig. 3: Steady-state $K_D$ approximation by biolayer interferometry (BLI).** **a** Representative interferograms showing the association (As.) and dissociation (Dis.) of soluble recombinant RABV G ectodomains to immobilized RVC20 variants at pH 8. Data were corrected for baseline drift and nonspecific binding to the sensors. Nonlinear regressions of the BLI shifts after 20 min of association are shown to the right of each interferogram. The upper and lower horizontal lines indicate extrapolated maximal shifts and half-maximal shifts, respectively. Vertical black lines indicate the (systematically overestimated) approximate $K_D$ values. The vertical red line indicates the corresponding value for the unmutated reference system '$G_{Ecto}$ WT - RVC20 Mature' displayed in the upper left corner of the panel. **b** Comparison of approximated $K_D$ values between samples. Significant differences between the wild-type system and the $G_{Ecto}$ V230K mutant and the domain III may arise from differences in sample quality. The $K_D$ of the RVC20 UA lies outside the investigated range, likely close to $10^{-6}$ M. $n$ = 3 measurements of the same samples. Data are displayed as means ± s.d. Statistical analysis was performed using Tukey's test with $\alpha$=0.05. ****$P$ < 0.0001; ***$P$ < 0.001; **$P$ < 0.01; *$P$ < 0.05; ns, $P$ > 0.05. Source data are provided as a Source Data file.

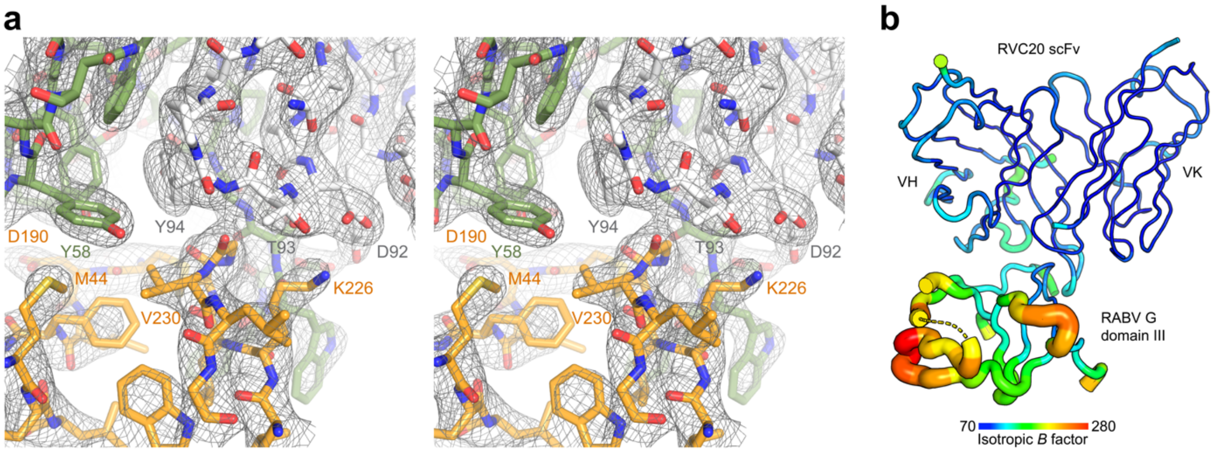


**Supplementary Fig. 4: Quality of the X-ray structure.**
**a** Stereo image of a portion of the crystallographic 2mFo-DFc electron density map contoured at 1.0 σ. **b** Isotropic atomic displacement factors (*B* factors) are color-coded for each residue with a gradient from blue (low) to red (high), indicating that although parts of the antigen located far from the mAb are poorly defined, the interface between the mAb and the antigen is well supported by the electron density map.

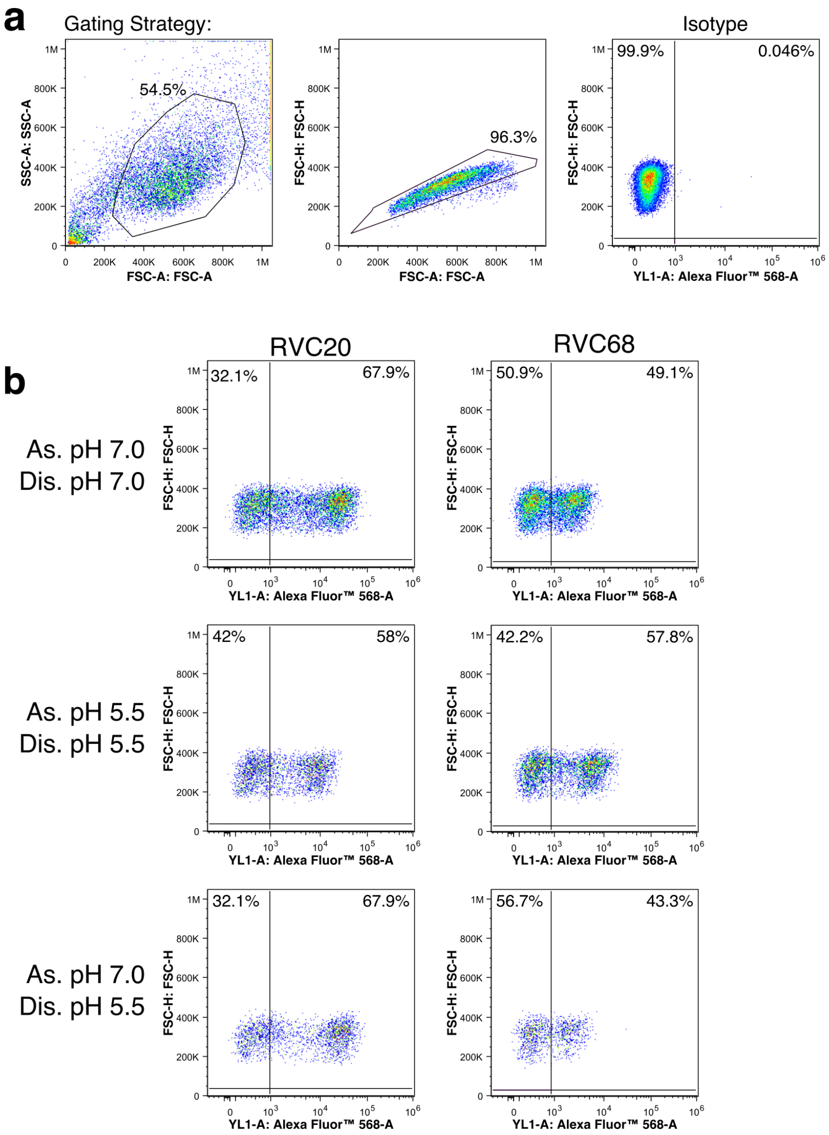


**Supplementary Fig. 5: Flow cytometry.**

**a** Gating strategy. Viable cells were gated based on forward and side scatter (left). Singlets were then gated using forward scatter height vs area (middle). AlexaFluor$^{TM}$ 568 positive and negative cell populations were defined using the isotype control stained cells as reference (right). **b** Pseudocolor dotplots of histograms shown in Fig. 3d.

**Supplementary Table 1: Primers for the amplification and mutagenesis of codon-optimized synthetic gene fragments[a]**

| Amplification | | Name | Sequence |
|---|---|---|---|
| pMT backbone for inserts with C-terminal single Strep tag[b,c] | 5′ | 2nd_Strep.Gf | GGTGGATGGTCACACCCTCAATTCGAGAAGT |
| | 3′ | BIP-REV | CCCGAGCGAGAGGCCAACAAA |
| pMT backbone for inserts without tag[c] | 5′ | pMT_TGA.Gf | TGAGTTTAAACCCGCTGATCAGCCTCGACTG |
| | 3′ | BIP-REV | CCCGAGCGAGAGGCCAACAAA |
| scFv RVC20-Strep[b] | 5′ | Bip.GF | CTGGCCGTCGTGGCCTTTGT |
| | 3′ | RVC20-SST.Gr | CTCGAATTGAGGGTGTGACCATCCACCCTTGATTTCCACCTTGGT |
| scFv RVC58[c] | 5′ | Bip.GF | CTGGCCGTCGTGGCCTTTGT |
| | 3′ | scFv58-TGA.Gr | GTCGAGGCTGATCAGCGGGTTTAAACTCACAGCACTGTCAGCTTTGT |
| RABV G domain III-Strep[b,c] | 5′ | RV_Bip-dIII.Gf | GCCTTTGTTGGCCTCTCGCTCGGGGAAGATGAGGGCTGCACCAAC |
| | 3′ | RV_del_dIV.Gr | GGTTCCCAGGCGTGGATTACCTCCACCCACTTTGATGCTGCTGATGTA |
| | 5′ | RV_del_dIV.Gf | ATCAGCAGCATCAAAGTGGGTGGAGGTAATCCACGCCTGGGAACCAGC |
| | 3′ | RV_dIII-SST.Gr | CTCGAATTGAGGGTGTGACCATCCACCATCATGCAGGTTGACCAGCTG |
| RABV G ecto-Strep[c] | 5′ | RABV_Bip-1.Gf | GCCTTTGTTGGCCTCTCGCTCGGGAAGTTCCCCATCTACACGATC |
| | 3′ | RABV_403-SST.Gr | CTCGAATTGAGGGTGTGACCATCCACCGCTTGGATCGGCCAGTGGATG |
| **Mutagenesis** | | **Name** | **Sequence** |
| RABV G ecto-Strep F74H[c] | 5′ | RABV_F74H.f | GCCGAGACATACACCAACCACGTGGGCTACGTGACCACC |
| | 3′ | RABV_F74H.r | GGTGGTCACGTAGCCCACGTGGTTGGTGTATGTCTCGGC |
| RABV G ecto-Strep W121H[c] | 5′ | RABV_W121H.f | CCCTATCCGGATTACCACCACCTGCGCACCGTCAAGAC |
| | 3′ | RABV_W121H.r | GTCTTGACGGTGCGCAGGTGGTGGTAATCCGGATAGGG |
| RABV G ecto-Strep D190S[c] | 5′ | RV_D190S.f | CGCCTGGGAACCAGCTGCAGCATCTTCACCAATAGCCGC |
| | 3′ | RV_D190S.r | GCGGCTATTGGTGAAGATGCTGCAGCTGGTTCCCAGGCG |
| RABV G ecto-Strep K226T[c] | 5′ | RV_K226T.f | AAGGGCGCCTGCAAGCTGACCCTGTGCGGAGTGTTGGGA |
| | 3′ | RV_K226T.r | TCCCAACACTCCGCACAGGGTCAGCTTGCAGGCGCCCTT |
| RABV G ecto-Strep V230M[c] | 5′ | RV_V230M.f | AAGCTGAAGCTGTGCGGAATGTTGGGACTGCGCCTGATG |
| | 3′ | RV_V230M.r | CATCAGGCGCAGTCCCAACATTCCGCACAGCTTCAGCTT |
| RABV G ecto-Strep V230K[c] | 5′ | RV_V230K.f | AAGCTGAAGCTGTGCGGAAAGTTGGGACTGCGCCTGATG |
| | 3′ | RV_V230K.r | CATCAGGCGCAGTCCCAACTTTCCGCACAGCTTCAGCTT |

[a] All cloning was performed by Gibson assembly (New England Biolabs)[2].

[b] To generate constructs used in crystallization.

[c] To generate constructs used in BLI.

**Supplementary Table 2: Primers for the amplification and mutagenesis of wild-type G[a]**

| Amplification | | Name | Sequence |
|---|---|---|---|
| phCMV | 5′ | pHCMV for | CAGGGAGGAGCTCGTGAACC |
| backbone[b] | 3′ | pHCMV rev | GGCTCTTCAGGAGTGGCCCACG |
| RABV G[b] | 5′ | G8743-HCMV for | CACTCCTGAAGAGCCATGATTCCTCAGGCTC |
| | 3′ | G8743-HCMV rev | CGAGCTCCTCCCTGCTACAGCTTGGTCTCAC |
| **Mutagenesis** | | **Name** | **Sequence** |
| RABV G D190S[c] | 5′ | D190S forward | GGACCTCTTGTTCGATTTTCACCAATAGC |
| | 3′ | D190S reverse | CTAGTCTCGGATTTTCTGGCATCCAGATAG |
| RABV G K226T[c] | 5′ | K226T forward | GGCATGCAAATTGACGCTATGTGGAG |
| | 3′ | K226T reverse | CCTTTTAGAGACCTGTACAAGCC |
| RABV G V230M[c] | 5′ | V230M forward | GAAACTATGTGGAATGCTTGGACTTAGAC |
| | 3′ | V230M/K reverse | AATTTGCATGCCCCTTTTAGAGACC |
| RABV G V230K[c] | 5′ | V230K forward | GAAACTATGTGGAAAGCTTGGACTTAGAC |
| | 3′ | V230M/K reverse | AATTTGCATGCCCCTTTTAGAGACC |

[a] Cloning was performed by In-Fusion cloning (Clontech), and mutagenesis was performed using the Phusion Site-Directed Mutagenesis Kit (Thermo Scientific).
[b] To generate constructs used in cell-cell fusion.
[c] To generate constructs used in reverse genetics.

## Supplementary References


1. Roche, S., Rey, F. A., Gaudin, Y. & Bressanelli, S. Structure of the Prefusion Form of the Vesicular Stomatitis Virus Glycoprotein G. *Science* **315**, 843–848 (2007).
2. Gibson, D. G. *et al.* Enzymatic assembly of DNA molecules up to several hundred kilobases. *Nat. Methods* **6**, 343–345 (2009).
