# Supplementary material for: Structure of the prefusion-locking broadly neutralizing antibody RVC20 bound to the rabies virus glycoprotein
Source: Nat Commun. 2020 Jan 30;11:596. doi: 10.1038/s41467-020-14398-7 (PMC6992781; doi:10.1038/s41467-020-14398-7)
Supplement: Supplementary file 3 — Reporting Summary [file 41467_2020_14398_MOESM3_ESM.pdf]

nature research

Corresponding author(s): Félix A. Rey

Last updated by author(s): Dec 20, 2019

Reporting Summary

Nature Research wishes to improve the reproducibility of the work that we publish. This form provides structure for consistency and transparency in reporting. For further information on Nature Research policies, see Authors & Referees and the Editorial Policy Checklist .

Statistics

For all statistical analyses, confirm that the following items are present in the figure legend, table legend, main text, or Methods section.

n/a	Confirmed	
☐	☒	The exact sample size (n) for each experimental group/condition, given as a discrete number and unit of measurement
☐	☒	A statement on whether measurements were taken from distinct samples or whether the same sample was measured repeatedly
☐	☒	The statistical test(s) used AND whether they are one- or two-sided<br>*Only common tests should be described solely by name; describe more complex techniques in the Methods section.*
☒	☐	A description of all covariates tested
☐	☒	A description of any assumptions or corrections, such as tests of normality and adjustment for multiple comparisons
☐	☒	A full description of the statistical parameters including central tendency (e.g. means) or other basic estimates (e.g. regression coefficient) AND variation (e.g. standard deviation) or associated estimates of uncertainty (e.g. confidence intervals)
☐	☒	For null hypothesis testing, the test statistic (e.g. F, t, r) with confidence intervals, effect sizes, degrees of freedom and P value noted<br>*Give P values as exact values whenever suitable.*
☒	☐	For Bayesian analysis, information on the choice of priors and Markov chain Monte Carlo settings
☒	☐	For hierarchical and complex designs, identification of the appropriate level for tests and full reporting of outcomes
☒	☐	Estimates of effect sizes (e.g. Cohen's d, Pearson's r), indicating how they were calculated

Our web collection on statistics for biologists contains articles on many of the points above.

Software and code

Policy information about availability of computer code

Data collection	X-ray data collection:<br>MXCuBE2 (http://mxcube.github.io/mxcube/)<br><br>Structure determination:<br>XDS June 1, 2017 (http://xds.mpimf-heidelberg.mpg.de/)<br>Global Phasing Limited STARANISO server release v3.201 (http://staraniso.globalphasing.org/)<br>Phenix 1.10.1-2155 (https://www.phenix-online.org/)<br>Coot 0.8.8-pre EL (https://www2.mrc-lmb.cam.ac.uk/personal/pemsley/coot/)<br><br>Biolayer Interferometry:<br>ForteBio Octet Data Acquisition 10.0 (https://www.fortebio.com/)<br><br>Opera imaging:<br>Harmony High-Content Imaging Software v4.8 (https://www.perkinelmer.com/)<br><br>Flow cytometry:<br>Attune NxT Software v2.7.0 (https://www.thermofisher.com/)
Data analysis	Structure analysis:<br>Dali web server (http://ekhidna.biocenter.helsinki.fi/dali_server/)<br>PDBePISA 1.52 web server (http://www.ebi.ac.uk/pdbe/pisa/)<br>Phyre2 web server (http://www.sbg.bio.ic.ac.uk/phyre2/)<br>PyMOL 2.4.0a0 (https://pymol.org/)

Sequence alignments and conservation analysis:
Clustal
Opera data analysis:
Columbus Image Data Storage and Analysis System v2.7 (https://www.perkinelmer.com/)

FACS data analysis:
Flowjo v9.3.2 (https://www.flowjo.com/)Omega web server (https://www.ebi.ac.uk/Tools/msa/clustalo/)
ESPript 3.0 web server (http://espript.ibcp.fr/)
IMGT/V-QUEST web server (http://www.imgt.org/IMGT_vquest/share/textes/)
R BioStrings (https://www.rdocumentation.org/)
R ggseqlogo (https://www.rdocumentation.org/)

Biolayer interferometry:
ForteBio Octet Data Analysis HT 10.0 (https://www.fortebio.com/)

Opera data analysis:
Columbus Image Data Storage and Analysis System v2.7 (https://www.perkinelmer.com/)

Flow cytometry:
Flowjo v9.3.2 (https://www.flowjo.com/)

Statistical analysis:
GraphPad Prism 6.0h (https://www.graphpad.com/)

For manuscripts utilizing custom algorithms or software that are central to the research but not yet described in published literature, software must be made available to editors/reviewers. We strongly encourage code deposition in a community repository (e.g. GitHub). See the Nature Research guidelines for submitting code & software for further information.

Data

Policy information about availability of data

All manuscripts must include a data availability statement. This statement should provide the following information, where applicable:

- Accession codes, unique identifiers, or web links for publicly available datasets
- A list of figures that have associated raw data
- A description of any restrictions on data availability

The authors declare that the data supporting the findings of this study are available within the paper and the Supplementary Information. The crystal structure of the RVC20 scFv / RABV G domain III complex from this study is available in the PDB with the accession code: 6TOU. The source data underlying Figs 1e, 1f, 2c, 3d, 3e and Supplementary Figure 3 are provided as a Source Data file.

Field-specific reporting

Please select the one below that is the best fit for your research. If you are not sure, read the appropriate sections before making your selection.

[x] Life sciences [ ] Behavioural & social sciences [ ] Ecological, evolutionary & environmental sciences

For a reference copy of the document with all sections, see nature.com/documents/nr-reporting-summary-flat.pdf

Life sciences study design

All studies must disclose on these points even when the disclosure is negative.

Sample size	No sample size calculations were performed.
Data exclusions	No data were excluded.
Replication	All experiments could be repeated successfully.
Randomization	No experiments presented in this study required randomization.
Blinding	No experiments presented in this study required blinding.

Reporting for specific materials, systems and methods

We require information from authors about some types of materials, experimental systems and methods used in many studies. Here, indicate whether each material, system or method listed is relevant to your study. If you are not sure if a list item applies to your research, read the appropriate section before selecting a response.

Materials & experimental systems

n/a	Involved in the study
☐	☒ Antibodies
☐	☒ Eukaryotic cell lines
☒	☐ Palaeontology
☒	☐ Animals and other organisms
☒	☐ Human research participants
☒	☐ Clinical data

Methods

n/a	Involved in the study
☒	☐ ChIP-seq
☐	☒ Flow cytometry
☒	☐ MRI-based neuroimaging

Antibodies

Antibodies used	Recombinant human IgG1 RVC20, and variants thereof as described in Fig. 2<br>Recombinant human IgG1 RVC68<br>Anti-Rabies nucoeocapsid conjugate - Bio-Rad - Cat# 3572112
Validation	Unmutated RABV G specific antibodies recognized their antigen in ELISA.<br>The commercial antibody was validated by the manufacturer.

Eukaryotic cell lines

Policy information about cell lines

Cell line source(s)	Drosophila S2 cells - Thermo Fisher Scientific - Cat# R69007<br>ExpiCHO cells - Thermo Fisher Scientific - Cat# A29127<br>BHK-21 clone 13 cells (ATCC CCL-10)<br>HEK 293T clone 17 cells (ATCC CRL-11268)<br>BSR cells (a BHK-21 clone, kindly provided by Monique Lafon, Institut Pasteur, Paris)<br>BSR-T7 cells (kindly provided by Karl-Klaus Conzelmann, Max von Pettenkofer Institute and Gene Center, Munich)
Authentication	The cell lines were not authenticated.
Mycoplasma contamination	All cell lines were tested negative for mycoplasma contamination.
Commonly misidentified lines<br>(See ICLAC register)	No commonly misidentified cell lines were used in this study.

Flow Cytometry

Plots

Confirm that:

- ☒ The axis labels state the marker and fluorochrome used (e.g. CD4-FITC).
- ☒ The axis scales are clearly visible. Include numbers along axes only for bottom left plot of group (a 'group' is an analysis of identical markers).
- ☐ All plots are contour plots with outliers or pseudocolor plots.
- ☒ A numerical value for number of cells or percentage (with statistics) is provided.

Methodology

Sample preparation	2x10^6 HEK293-T cells were transfected in suspension with 100 ng phCMV-8743 mixed with 1900 ng pQCXIP-empty using Lipofectamine 2000 (Thermo Fisher). 18 h after transfection, the cells were lifted using PBS + 0.1% EDTA. The cells were subsequently resuspended in 60 mM MES + 100 mM NaCl at pH 7.0 or pH 5.5, and were equilibrated for 15 min at 37°C, shaking at 1100 rpm to avoid cell-cell fusion. Equilibration was followed by a mAb association step at 800 ng ml^-1 of isotype control mGO53, RVC20, or RVC68 in the indicated buffers for 15 min at room temperature with shaking. The cells were next washed in the indicated buffers and incubated for an additional 15 min shaking in the same buffers. After two washing steps in PBS, the cells were stained with secondary Goat anti-Human IgG, Alexa Fluor 568 (Invitrogen, #A21090) diluted 1:500 in PBS + 1% BSA. Cells were washed and fixed for 10 minutes in 4% PFA prior to fluorescence measurement on an Attune NxT Flow Cytometer (Thermo Fisher). The gating strategy is exemplified in Supplementary Figure 5a.
Instrument	Attune NxT Flow Cytometer; Model: 4486521 Attune NxT Acoustic Focusing Cytometer (Laser: BRVY)
Software	Flowjo (9.3.2)

Cell population abundance	RABVg transfected cells (AlexaFluor 568 postive cells) represented 62±11 % of the FSC-A/FSC-H parental gate under pH 7 staining conditions.
Gating strategy	Viable cells were gated using FSC-A/SSC-A. Doublets were removed by using the FSC-H/FSC-A gate. Boundary between AlexaFluor 568 positive and negative cells was defined based on the isotype control (Supplementary Figure 5a).

☒ Tick this box to confirm that a figure exemplifying the gating strategy is provided in the Supplementary Information.
